# New insights into retroviral Gag–Gag and Gag–membrane interactions

**DOI:** 10.3389/fmicb.2014.00302

**Published:** 2014-06-24

**Authors:** José O. Maldonado, Jessica L. Martin, Joachim D. Mueller, Wei Zhang, Louis M. Mansky

**Affiliations:** ^1^Institute for Molecular Virology, University of MinnesotaMinneapolis, MN, USA; ^2^Department of Diagnostic and Biological Sciences, School of Dentistry, University of Minnesota, Minneapolis, MN, USA; ^3^Pharmacology Graduate Program, University of MinnesotaMinneapolis, MN, USA; ^4^School of Physics and Astronomy, University of MinnesotaMinneapolis, MN, USA; ^5^Characterization Facility, University of MinnesotaMinneapolis, MN, USA; ^6^Department of Microbiology, University of MinnesotaMinneapolis, MN, USA

**Keywords:** plasma membrane, oligomerization, multimerization, lentivirus, deltaretrovirus, spectroscopy

## Abstract

A critical aspect of viral replication is the assembly of virus particles, which are subsequently released as progeny virus. While a great deal of attention has been focused on better understanding this phase of the viral life cycle, many aspects of the molecular details remain poorly understood. This is certainly true for retroviruses, including that of the human immunodeficiency virus type 1 (HIV-1; a lentivirus) as well as for human T-cell leukemia virus type 1 (HTLV-1; a deltaretrovirus). This review discusses the retroviral Gag protein and its interactions with itself, the plasma membrane and the role of lipids in targeting Gag to virus assembly sites. Recent progress using sophisticated biophysical approaches to investigate – in a comparative manner – retroviral Gag–Gag and Gag–membrane interactions are discussed. Differences among retroviruses in Gag–Gag and Gag–membrane interactions imply dissimilar molecular aspects of the viral assembly pathway, including the interactions of Gag with lipids at the membrane.

The assembly of virus particles is a key aspect of viral replication that is still poorly understood at the molecular level. Retroviral assembly has been extensively investigated, though detailed information is lacking for many aspects of the process. This is true for human immunodeficiency virus type 1 (HIV-1), and particularly true for human T-cell leukemia virus type 1 (HTLV-1). In this review, we review the retroviral Gag protein, its translocation to the plasma membrane (PM), as well as Gag–Gag and Gag–PM interactions. We then highlight recent progress made using sophisticated biophysical approaches to investigate Gag–Gag and Gag–PM interactions, which represent key early events in the virus assembly pathway, including that of interacting with lipids at the PM.

## THE Gag POLYPROTEIN

**FIGURE 1:**
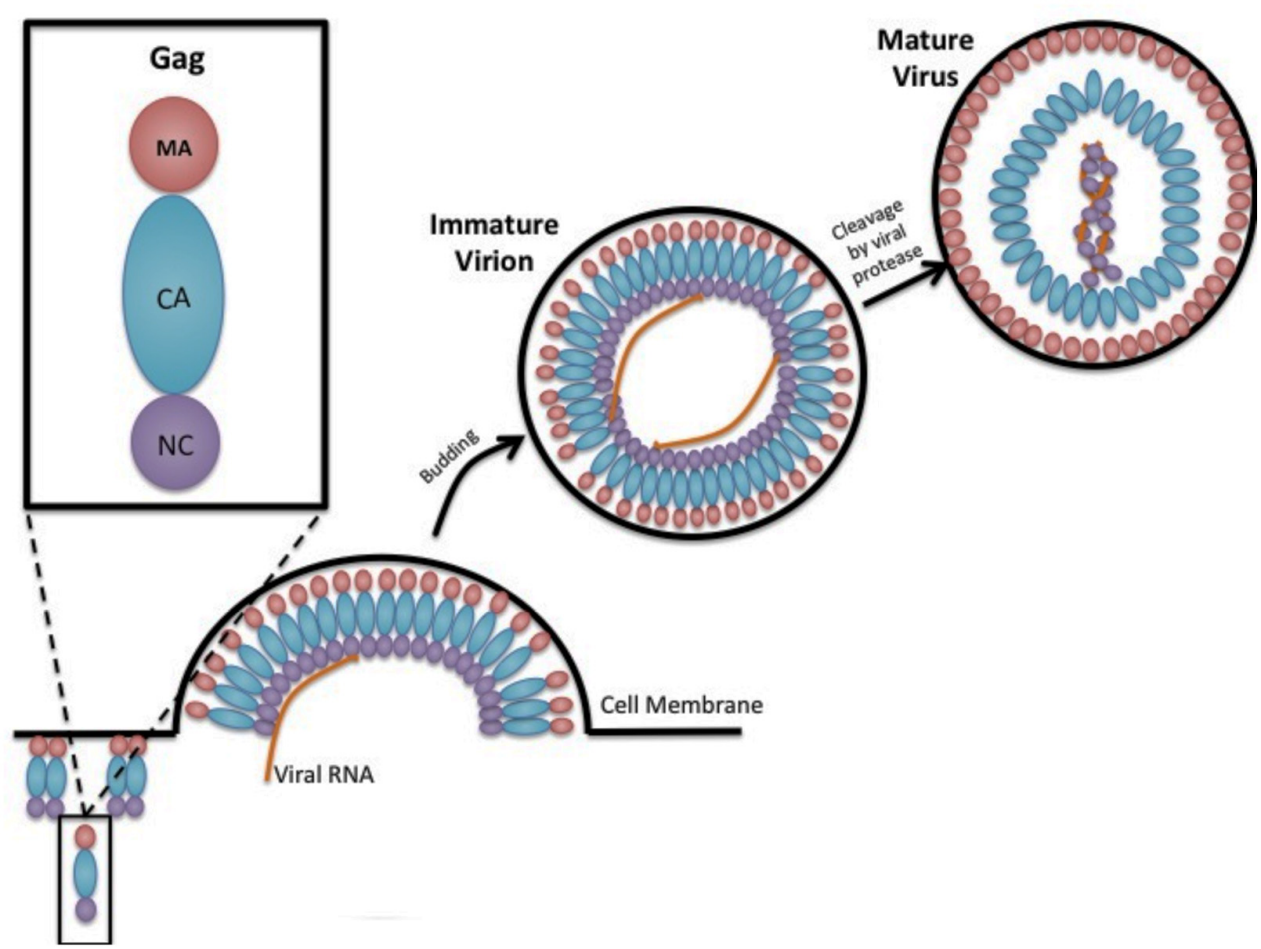
**Gag and retrovirus particle assembly.** A cartoon depiction is shown in cross section of the assembly of a prototypic retrovirus particle, emphasizing the oligomerization of Gag along the inner leaflet of the plasma membrane, incorporation of two copies of the viral RNA, the budding of immature virus particles, and the conversion of the immature virus particle to mature infectious virus particle that is catalyzed by the viral-encoded protease. Gag is shown as being composed of the matrix domain (red circle), the capsid domain (blue oval), and the nucleocapsid domain (purple circle). Two copies of the viral RNA (two orange lines inside the viral particle) are shown packaged into the virus particle.

Gag is the primary retroviral structural protein responsible for orchestrating the majority of steps in viral assembly. Most of these assembly steps occur via interactions with three Gag subdomains – matrix (MA), capsid (CA), and nucleocapsid (NC; **Figure 1**). These three regions have a low level of sequence conservation among the different retroviral genera, which belies the observed high level of structural conservation. Outside of these three domains, Gag proteins can vary widely. For example, HIV-1 Gag additionally codes for a C-terminal p6 protein as well as two spacer proteins, SP1 and SP2, which demarcate the CA–NC and NC–p6 junctions, but HTLV-1 contains no additional sequences outside of MA, CA, and NC (Oroszlan and Copeland, 1985; Henderson et al., 1992).

The Gag subdomains are structurally discrete but have functionally overlapping roles in the viral assembly process. The N-terminus of Gag begins with MA, which contains key residues responsible for the recruitment of Gag to the PM via an N-terminal myristoyl moiety and a highly basic region (Bryant and Ratner, 1990; Zhou et al., 1994; Ono et al., 2000; Stansell et al., 2007; Hamard-Peron et al., 2010). CA is divided into two structurally distinct domains – the N-terminal domain (NTD) and C-terminal domain (CTD) – and contains the majority of the residues responsible for Gag–Gag interactions. While the primary amino acids for HIV-1 Gag oligomerization are located in the CA CTD (Dorfman et al., 1994; Gamble et al., 1997; Alfadhli et al., 2005; Dalton et al., 2007; Datta et al., 2007), there are additional residues located throughout CA, NC, and SP1 that are responsible for laterally stabilizing Gag–Gag interactions (Krausslich et al., 1995; von Schwedler et al., 2003; Li et al., 2007). Functionally distinct from the CTD, in HIV-1 the CA NTD is not necessary for viral assembly (Borsetti et al., 1998; Accola et al., 2000). Finally, NC possesses two zinc finger domains along with several key amino acids that function to bind and package viral RNA into particles (Gorelick et al., 1988; Cimarelli et al., 2000; Muriaux et al., 2004). It is important to note that the functions of these domains are not exclusive, and there is much overlap. For example, HIV-1 NC has recently been implicated in facilitating the budding process, which was believed to be driven solely by motifs within the p6 domain (Dussupt et al., 2011; Bello et al., 2012).

Once a cell has been infected by a retrovirus, full-length Gag polyproteins assemble to form immature virions. The reported numbers of Gag incorporated in each particle varies greatly from ∼750–5000 (Yeager et al., 1998; Vogt and Simon, 1999; Briggs et al., 2004; Chen et al., 2009). It is theorized that the preparation of the particles can affect this number as well as the genus of retrovirus. Upon the assembly of these Gag proteins into a lattice shell, the viral protease will cleave the Gag polyprotein into its respective domains to form a mature virus. This cleavage event changes the virus morphology, yielding a CA core, which is conical in the case of HIV-1 (Ganser et al., 1999; Briggs and Krausslich, 2011). In addition to the change in morphology, the individual Gag domain proteins no longer exhibit the same roles as the full-length Gag polyprotein.

## Gag TRAFFICKING TO THE PLASMA MEMBRANE

Previously, retroviral research posited that Gag proteins trafficked to endosomal compartments and assembled there. This model was based on studies that found Gag in intracellular endosomal compartments (Raposo et al., 2002; Nydegger et al., 2003; Pelchen-Matthews et al., 2003) as well as studies that found virions associated with the mannose receptor CD63 (Nguyen and Hildreth, 2003; Ono et al., 2004; Gousset et al., 2008). Over the past decade, this idea has been challenged by studies showing that the vacuolar structures bearing retroviral virions were actually invaginations of the PM (Welsch et al., 2007; Finzi et al., 2013). Additionally, it was found that HIV-1 Gag is only found in endosomes at late time points and preventing endosomal function with drugs does not inhibit the release of viral particles (Jouvenet et al., 2006; Finzi et al., 2007). Based upon these and other studies, it is generally agreed that the productive site of retroviral particle assembly is the PM.

Before particle assembly can occur at the PM, Gag must traffic from the site of synthesis through the cytoplasm. While this process is not well-understood, it is increasingly thought that Gag must interact with a large amount of host cell machinery, including microtubule networks, motor proteins, and vacuolar transport complexes.

Microtubules are highly dynamic structures that make up the cytoskeleton of the cell. They serve as the road-like structures that motor proteins such as kinesins use to traffic cellular cargo to various cytoplasmic locations (Vale, 2003). It has been shown that HIV-1 Gag interacts with kinesin superfamily member KIF4, a protein that traffics cellular cargo from the perinucleolar region to the membrane (Hirokawa and Noda, 2008). Downregulating KIF4 not only slows Gag trafficking and reduces particle production but also seemingly increases intracellular Gag degradation, indicating that KIF4 has some sort of stabilizing action. Additionally, suppressor of cytokine signaling 1 (SOCS1) has been shown to colocalize with Gag along the cytoskeleton and promote microtubule stability (Nishi et al., 2009). These studies would indicate that Gag binds to host cell proteins to stabilize its transport via cytoskeletal networks.

The idea that Gag utilizes the microtubule network to directly traffic to the PM is not without controversy (Naghavi and Goff, 2007). Other studies have shown that kinesin family members that direct membranous organelles such as endosomes and lysosomes to the PM are involved in HIV-1 replication (Brass et al., 2008; Konig et al., 2008; Zhou et al., 2008). Studies have yet to reconcile these different pathways of Gag transport. It is likely that there are multiple trafficking pathways that lead to Gag expression at the PM, but further analyses need to be done to clarify the mechanisms.

## DETERMINANTS OF Gag–Gag INTERACTIONS

The formation of Gag–Gag oligomers appears to be a complex and multifactorial process involving several Gag domains, host proteins, and environmental factors. Examples of important host proteins include the molecular motors and microtubule networks mentioned above, which can concentrate Gag in particular locations of the cell (Naghavi and Goff, 2007). Gag concentration levels as well as subcellular location of Gag are some of the important environmental factors that can contribute to Gag oligomerization.

It is known that CA is the primary region involved in oligomerization (Gamble et al., 1997). In HIV-1, it has been shown that mutations specifically in the CA CTD affect Gag–Gag interactions and severely impede viral particle production (von Schwedler et al., 2003). Based on studies using chimeric Gag molecules with different CA domains, it appears that this is the case for other retroviruses as well (Ako-Adjei et al., 2005). In HTLV-1, the CA NTD is required in addition to the CTD to form Gag–Gag interactions (Rayne et al., 2001).

**FIGURE 2:**
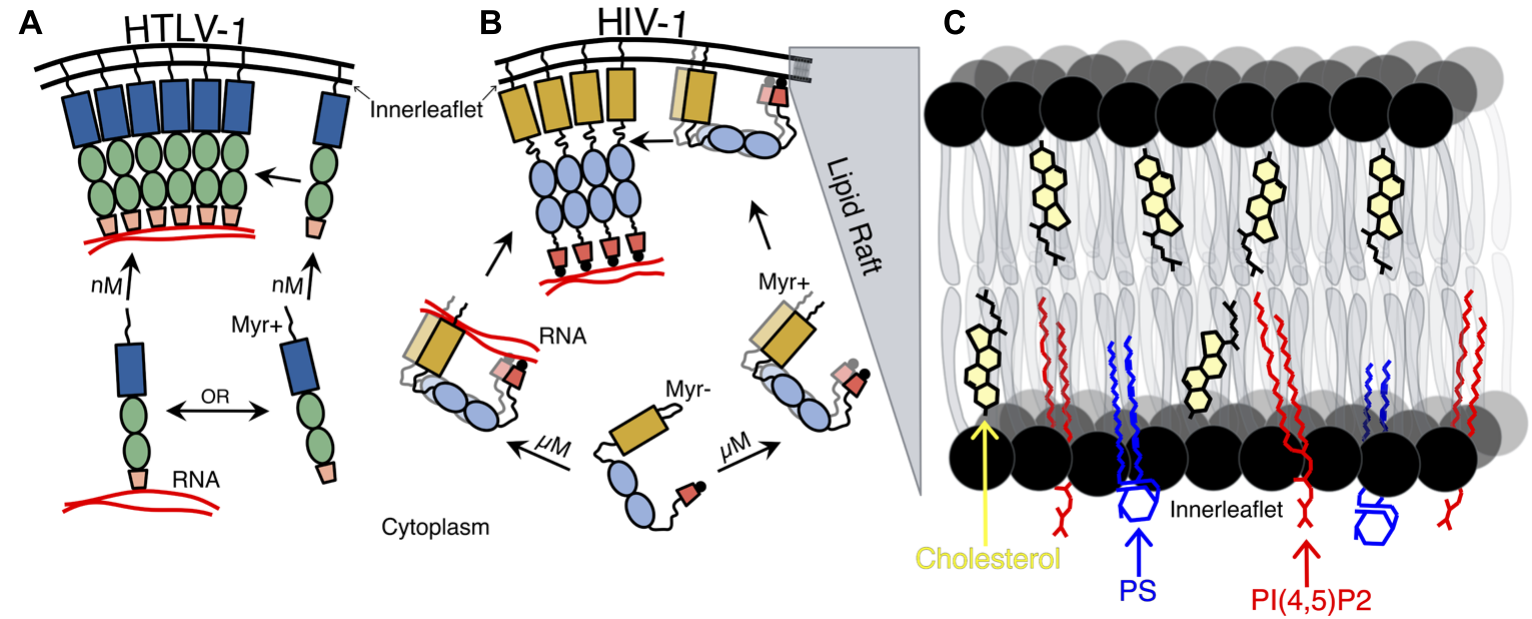
**Schematic representation of HTLV-1 and HIV-1 Gag– membrane association. (A)** HTLV-1 Gag associates with the plasma membrane (PM) as a monomer and is found in the PM at nM concentrations. HTLV-1 Gag is shown as recruiting viral RNA after its association with the inner leaflet of the PM (though monomeric Gag may recruit the viral RNA in the cytoplasm and are transported together to the PM). **(B)** Concentration- dependent HIV-1 Gag dimerization and translocation to the PM. HIV-1 Gag must reach a critical cytoplasmic concentration (∼0.5 μM) in order for Gag–Gag dimers to form and subsequent Gag–membrane association to occur. The myristoyl moiety of the HIV-1 Gag is exposed, allowing for association with the PM of Gag dimers (including Gag dimers associated with dimeric viral RNA). Both HIV-1 Gag N- and CTD interact with the inner leaflet of the PM and to NA, but is not until HIV-1 Gag interacts with the PM, other HIV-1 Gag molecules, and to NA that the protein becomes extended. **(C)** Expanded view of lipid raft that HIV-1 Gag associates with at the PM. Shown are three key constituents of lipid rafts: cholesterol (yellow), phosphatidylserine (PS; blue), and phosphatidylinositol 4,5-bisphosphate [PI(4,5)P2; red].

While CA is typically thought to be the primary site of Gag–Gag interactions, other Gag domains are necessary for stabilizing these interactions. NC has been shown to be an important factor in the formation of Gag–Gag interactions, likely due to its RNA-binding capacities. However, it has also been shown that the HIV-1 MA NTD is capable of binding to the RNA through an electrostatic interaction (Purohit et al., 2001). RNA may serve as a binding platform for Gag assembly, as it may promote Gag oligomerization and expose domains necessary for interactions with the PM (**Figures 2A,B**; Khorchid et al., 2002; Hogue et al., 2009; Rein et al., 2011).

Myristoylation appears to be another requirement for Gag–Gag multimerization, at least in the case of HIV-1 (Li et al., 2007; O’Carroll et al., 2012). Fluorescence resonance energy transfer (FRET) studies found that without a myristic acid moiety, there was a significant decrease in Gag–Gag interactions. The simplest explanation for this phenomenon is that myristoylation concentrates Gag molecules at the PM, bringing the CA domains into contact and facilitating oligomerization.

The requirement for the CA interface, nucleic acid scaffolding, and myristic moiety have repeatedly been confirmed (Li et al., 2007; O’Carroll et al., 2012, 2013). It appears that this “functional redundancy” allows for some give in the ability of HIV-1 to assemble – only two of the three components are necessary for VLP production – but when more than one function is ablated, no particles can be produced (O’Carroll et al., 2012).

## Gag OLIGOMERIZATION

Eventually, Gag must form higher order oligomers (e.g., dimers, trimers) in order to form the lattice structure seen in immature virions and virus-like particles. It is unlikely that these large-scale interactions form in the cytosol, as Gag is translated as a soluble protein. Gag–Gag interactions have been primarily studied using HIV-1 as a model system. This system has been thought to be useful due to the structural similarities between retroviruses, specifically the MA domain. Initial studies used crosslinking to show that HIV-1 Gag mainly exists as a dimer in the cytoplasm and does not form trimers or hexamers until it reaches the PM (Kutluay and Bieniasz, 2010). This observation was recently confirmed using dual-color z-scan fluorescence fluctuation spectroscopy (dczFFS), which quantifies fluorescent proteins inside of living cells (Fogarty et al., 2011, 2013).

It has typically been thought that other retroviruses, such as HTLV-1, trafficked to and assembled at the PM in a similar fashion to that of HIV-1. Using dcz-FFS together with total internal reflection fluorescence and conventional, epi-illumination imaging, it was recently reported that HIV-1 requires micromolar concentrations of Gag in order for it to target and associate with the PM (**Figure 2B**), while HTLV-1 only requires nanomolar concentrations of Gag to become associated with the PM (**Figure 2A**). These results correlate with previous observations that HTLV-1 Gag–Gag interactions were absent in the cytoplasm (Fogarty et al., 2011). This data also supports the hypothesis that HTLV-1 reaches the PM as a monomer where it then forms higher order oligomers (**Figure 2A**), which is in contrast to HIV-1 Gag–Gag interactions, which traffics as lower order oligomers in the cytoplasm prior to targeting the PM (**Figure 2B**; Lindwasser and Resh, 2001; Perez-Caballero et al., 2004). Therefore, HTLV-1 Gag monomers must translocate to the same area at the inner leaflet of the PM to form Gag–Gag oligomers (**Figure 2A**).

The facts that HTLV-1 NC is a bad chaperone (Qualley et al., 2010) and that HTLV-1 Gag–membrane interaction is independent of viral RNA binding (Inlora et al., 2011), taken together with the finding that HTLV-1 Gag targets the PM at low cytoplasmic concentrations (Fogarty et al., 2013), suggest that the viral RNA interacts with Gag at the PM, acting as a scaffold for Gag–Gag interactions (**Figure 2A**). However, it is possible that HTLV-1 monomers interact and traffic the viral RNA to the PM. In the case of HIV-1 Gag, it has been suggested that only a few Gag molecules are needed to recruit the viral RNA to the cytoplasm, which play an important role in initiating the assembly of HIV-1 virions at the PM (**Figure 2B**; Jouvenet et al., 2009). This indicates that different retroviruses may have different determinants of Gag–Gag interactions and Gag trafficking pathways, despite similarities in viral particle morphology.

## Gag–MEMBRANE INTERACTIONS

In order for Gag molecules to initiate particle assembly, it must recognize a site of assembly at the PM where it oligomerizes into higher-order multimers. It has been shown that Gag targets and assembles at specific PM microdomains known as lipid rafts, which are dense, ordered groups of tightly packed saturated lipids stabilized by cholesterol (**Figure 2C**). The molecular composition of these lipid rafts is different than that of the surrounding membrane (Lingwood et al., 2009; Ono, 2009; Sonnino and Prinetti, 2013).

MA is responsible for the binding of Gag to the inner leaflet of the PM, which is mediated by the MA NTD that contains multiple membrane binding signals necessary for membrane interactions. A hydrophobic myristic acid moiety found in MA of most retroviruses, such as HIV-1 and HTLV-1, plays an important role in targeting and inserting Gag to the inner leaflet of the PM (Ootsuyama et al., 1985; Zhou and Resh, 1996; Resh, 1999, 2004; Tang et al., 2004). MA also contains a highly basic region, mostly arginines and lysines, that interacts electrostatically with the inner leaflet of the PM. Furthermore, it has been shown that HIV-1 Gag is flexible and can adopt a closed conformation, which brings the MA and NC terminal domains in close proximity, allowing these to interact with the anionic inner leaflet of the PM (**Figure 2B**; Datta et al., 2011). The inner leaflet of the PM is rich in acidic phospholipids, such as phosphatidylserine (PS) and acidic phosphatidylinositol-4,5-bisphosphate [PI(4,5)P_2_], which is important for efficient membrane binding and targeting to the PM (**Figure 2C**; Murray et al., 2005; Chukkapalli et al., 2008).

## CHARACTERISTICS ALONG THE INNER LEAFLET OF THE PLASMA MEMBRANE THAT ARE SITE OF Gag ASSEMBLY

The PM is composed of different transmembrane proteins and a wide variety of lipids. These include cholesterol and multiple phospholipids such as phosphatidylinositol phosphates (PIPs), phosphatidyl glycerol (PG), and PS; however, the most abundant lipids are phosphatidylcholine (PC) and phosphatidylethanolamine (PE). The inner leaflet of the PM is mostly composed of PE, PC, PS, and PI(4,5)P_2_, which makes it acidic and better suited for MA to interact with the PM (van Meer et al., 2008; Chukkapalli and Ono, 2011). HIV-1 Gag uses each one of these lipids as a signal to recognize the PM. However, as one of the main lipids in the inner leaflet, it has been suggested that HIV-1 Gag primarily interacts with PI(4,5)P_2_ which promotes the HIV-1 Gag to preferentially target the PM (Ono et al., 2004). Further studies have contradicted these findings and suggest that PI(4,5)P_2_ is not the most important site-specific acidic signal in the PM for HIV-1 Gag, HTLV-1 and other retroviral Gag proteins (e.g., RSV Gag) that do not have a PI(4,5)P_2_ binding signal, but rather can strongly interact with other acidic phospholipids (Chan et al., 2011; Inlora et al., 2011). However, contradictory results show that RSV Gag does interact with PI(4,5)P_2_ at the PM (Nadaraia-Hoke et al., 2013). Furthermore, HIV-1 can differentiate membranes with multiple compositions of fatty acids and cholesterol (Dick et al., 2012).

## Gag MOVEMENT AND TRAFFICKING ALONG THE INNER LEAFLET

A new assembly model for HIV-1 suggests that the viral genome is recruited by Gag and then directed and anchored to the PM (Jouvenet et al., 2009). In the latter location, the RNA–Gag complex functions as a scaffold to form large Gag oligomers by recruiting other Gag molecules (**Figures 2A,B**). The lateral movement of the viral RNA in the PM and the progressive accumulation of Gag molecules over time, suggest that after the RNA–Gag complex attaches to the PM, it moves in the PM plane to recruit more Gag molecules. Furthermore, it has been shown that HIV-1 Gag translocate from internal compartments towards virological synapses, which are the contact sites that allow the virus to be transferred, between infected macrophages and uninfected T cells (Gousset et al., 2008). Therefore, it is possible that the Gag anchored to the PM can also traffic to these virological synapses. Further studies need to be done to fully understand Gag movement along the infer leaflet of the PM.

## CONCLUDING REMARKS

Recent observations have demonstrated differences in the form and concentration of Gag that is associated with translocation to the PM. While HIV-1 Gag dimers are the primary form of Gag that is thought to translocate to the PM at μM cytoplasmic concentrations (i.e., concentration dependent translocation), HTLV-1 Gag has been found to translocate to the PM as a monomer at nM cytoplasmic concentrations. These observations indicate that fundamental differences may exist in the association of different Gag proteins with the PM, including interactions with lipids. Furthermore, such differences suggest that the movement of Gag along the inner leaflet of the PM may also be distinct among different retroviruses. Taken together, these observations argue for the importance of comparative studies of retroviruses in order to provide the greatest insights into the diversity of strategies associated with the virus assembly pathway.

## Conflict of Interest Statement

The authors declare that the research was conducted in the absence of any commercial or financial relationships that could be construed as a potential conflict of interest.

## References

[B1] Accola M. A. Strack B. Gottlinger H. G. (2000). Efficient particle production by minimal Gag constructs which retain the carboxy-terminal domain of human immunodeficiency virus type 1 capsid-p2 and a late assembly domain. *J. Virol.* 74 5395–5402 10.1128/JVI.74.12.5395-5402.2000 10823843 PMC112023

[B2] Ako-Adjei D. Johnson M. C. Vogt V. M. (2005). The retroviral capsid domain dictates virion size, morphology, and coassembly of gag into virus-like particles. *J. Virol.* 79 13463–13472 10.1128/JVI.79.21.13463-13472.2005 16227267 PMC1262573

[B3] Alfadhli A. Dhenub T. C. Still A. Barklis E. (2005). Analysis of human immunodeficiency virus type 1 Gag dimerization-induced assembly. *J. Virol.* 79 14498–14506 10.1128/JVI.79.23.14498-14506.2005 16282449 PMC1287545

[B4] Bello N. F. Dussupt V. Sette P. Rudd V. Nagashima K. Bibollet-Ruche F. (2012). Budding of retroviruses utilizing divergent L domains requires nucleocapsid. *J. Virol.* 86 4182–4193 10.1128/JVI.07105-11 22345468 PMC3318634

[B5] Borsetti A. Ohagen A. Gottlinger H. G. (1998). The C-terminal half of the human immunodeficiency virus type 1 Gag precursor is sufficient for efficient particle assembly. *J. Virol.* 72 9313–9317 9765481 10.1128/jvi.72.11.9313-9317.1998 PMC110353

[B6] Brass A. L. Dykxhoorn D. M. Benita Y. Yan N. Engelman A. Xavier R. J. (2008). Identification of host proteins required for HIV infection through a functional genomic screen. *Science* 319 921–926 10.1126/science.1152725 18187620

[B7] Briggs J. A. Krausslich H. G. (2011). The molecular architecture of HIV. *J. Mol. Biol.* 410 491–500 10.1016/j.jmb.2011.04.021 21762795

[B8] Briggs J. A. Simon M. N. Gross I. Krausslich H. G. Fuller S. D. Vogt V. M. (2004). The stoichiometry of Gag protein in HIV-1. *Nat. Struct. Mol. Biol.* 11 672–675 10.1038/nsmb785 15208690

[B9] Bryant M. Ratner L. (1990). Myristoylation-dependent replication and assembly of human immunodeficiency virus 1. *Proc. Natl. Acad. Sci. U.S.A.* 87 523–527 10.1073/pnas.87.2.523 2405382 PMC53297

[B10] Chan J. Dick R. A. Vogt V. M. (2011). Rous sarcoma virus gag has no specific requirement for phosphatidylinositol-(45)-bisphosphate for plasma membrane association in vivo or for liposome interaction in vitro. *J. Virol.* 85 10851–10860 10.1128/JVI.00760-11 21813603 PMC3187501

[B11] Chen Y. Wu B. Musier-Forsyth K. Mansky L. M. Mueller J. D. (2009). Fluorescence fluctuation spectroscopy on viral-like particles reveals variable gag stoichiometry. *Biophys. J.* 96 1961–1969 10.1016/j.bpj.2008.10.067 19254556 PMC2717261

[B12] Chukkapalli V. Hogue I. B. Boyko V. Hu W. S. Ono A. (2008). Interaction between the human immunodeficiency virus type 1 Gag matrix domain and phosphatidylinositol-(45)-bisphosphate is essential for efficient gag membrane binding. *J. Virol.* 82 2405–2417 10.1128/JVI.01614-07 18094158 PMC2258911

[B13] Chukkapalli V. Ono A. (2011). Molecular determinants that regulate plasma membrane association of HIV-1 Gag. *J. Mol. Biol.* 410 512–524 10.1016/j.jmb.2011.04.015 21762797 PMC3139151

[B14] Cimarelli A. Sandin S. Hoglund S. Luban J. (2000). Basic residues in human immunodeficiency virus type 1 nucleocapsid promote virion assembly via interaction with RNA. *J. Virol.* 74 3046–3057 10.1128/JVI.74.7.3046-3057.2000 10708419 PMC111803

[B15] Dalton A. K. Ako-Adjei D. Murray P. S. Murray D. Vogt V. M. (2007). Electrostatic interactions drive membrane association of the human immunodeficiency virus type 1 Gag MA domain. *J. Virol.* 81 6434–6445 10.1128/JVI.02757-06 17392361 PMC1900125

[B16] Datta S. A. Heinrich F. Raghunandan S. Krueger S. Curtis J. E. Rein A. (2011). HIV-1 Gag extension: conformational changes require simultaneous interaction with membrane and nucleic acid. *J. Mol. Biol.* 406 205–214 10.1016/j.jmb.2010.11.051 21134384 PMC3046808

[B17] Datta S. A. Zhao Z. Clark P. K. Tarasov S. Alexandratos J. N. Campbell S. J. (2007). Interactions between HIV-1 Gag molecules in solution: an inositol phosphate-mediated switch. *J. Mol. Biol.* 365 799–811 10.1016/j.jmb.2006.10.072 17098251 PMC1829305

[B18] Dick R. A. Goh S. L. Feigenson G. W. Vogt V. M. (2012). HIV-1 Gag protein can sense the cholesterol and acyl chain environment in model membranes. *Proc. Natl. Acad. Sci. U.S.A.* 109 18761–18766 10.1073/pnas.1209408109 23010924 PMC3503231

[B19] Dorfman T. Bukovsky A. Ohagen A. Hoglund S. Gottlinger H. G. (1994). Functional domains of the capsid protein of human immunodeficiency virus type 1. *J. Virol.* 68 8180–8187 7966609 10.1128/jvi.68.12.8180-8187.1994 PMC237283

[B20] Dussupt V. Sette P. Bello N. F. Javid M. P. Nagashima K. Bouamr F. (2011). Basic residues in the nucleocapsid domain of Gag are critical for late events of HIV-1 budding. *J. Virol.* 85 2304–2315 10.1128/JVI.01562-10 21159863 PMC3067763

[B21] Finzi A. Orthwein A. Mercier J. Cohen E. A. (2007). Productive human immunodeficiency virus type 1 assembly takes place at the plasma membrane. *J. Virol.* 81 7476–7490 10.1128/JVI.00308-07 17507489 PMC1933344

[B22] Finzi A. Perlman M. Bourgeois-Daigneault M. C. Thibodeau J. Cohen E. A. (2013). Major histocompatibility complex class-II molecules promote targeting of human immunodeficiency virus type 1 virions in late endosomes by enhancing internalization of nascent particles from the plasma membrane. *Cell. Microbiol.* 15 809–822 10.1111/cmi.12074 23170932 PMC3955195

[B23] Fogarty K. H. Berk S. Grigsby I. F. Chen Y. Mansky L. M. Mueller J. D. (2013). Interrelationship between cytoplasmic retroviral Gag concentration and Gag–membrane association. *J. Mol. Biol*. 426 1611–1624 10.1016/j.jmb.2013.11.025 24316368 PMC3951590

[B24] Fogarty K. H. Chen Y. Grigsby I. F. Macdonald P. J. Smith E. M. Johnson J. L. (2011). Characterization of cytoplasmic Gag–gag interactions by dual-color z-scan fluorescence fluctuation spectroscopy. *Biophys. J.* 100 1587–1595 10.1016/j.bpj.2011.02.008 21402042 PMC3059735

[B25] Gamble T. R. Yoo S. Vajdos F. F. von Schwedler U. K. Worthylake D. K. Wang H. (1997). Structure of the carboxyl-terminal dimerization domain of the HIV-1 capsid protein. *Science* 278 849–853 10.1126/science.278.5339.849 9346481

[B26] Ganser B. K. Li S. Klishko V. Y. Finch J. T. Sundquist W. I. (1999). Assembly and analysis of conical models for the HIV-1 core. *Science* 283 80–83 10.1126/science.283.5398.80 9872746

[B27] Gorelick R. J. Henderson L. E. Hanser J. P. Rein A. (1988). Point mutants of Moloney murine leukemia virus that fail to package viral RNA: evidence for specific RNA recognition by a “zinc finger-like” protein sequence. *Proc. Natl. Acad. Sci. U.S.A.* 85 8420–8424 10.1073/pnas.85.22.8420 3141927 PMC282469

[B28] Gousset K. Ablan S. D. Coren L. V. Ono A. Soheilian F. Nagashima K. (2008). Real-time visualization of HIV-1 GAG trafficking in infected macrophages. *PLoS Pathog.* 4:e1000015 10.1371/journal.ppat.1000015 PMC2267008 18369466

[B29] Hamard-Peron E. Juillard F. Saad J. S. Roy C. Roingeard P. Summers M. F. (2010). Targeting of murine leukemia virus gag to the plasma membrane is mediated by PI(45)P2/PS and a polybasic region in the matrix. *J. Virol.* 84 503–515 10.1128/JVI.01134-09 19828619 PMC2798412

[B30] Henderson L. E. Bowers M. A. Sowder R. C. II Serabyn S. A. Johnson D. G. Bess J. W. (1992). Gag proteins of the highly replicative MN strain of human immunodeficiency virus type 1: posttranslational modifications, proteolytic processings, and complete amino acid sequences. *J. Virol.* 66 1856–1865 1548743 10.1128/jvi.66.4.1856-1865.1992 PMC288972

[B31] Hirokawa N. Noda Y. (2008). Intracellular transport and kinesin superfamily proteins, KIFs: structure, function, and dynamics. *Physiol. Rev.* 88 1089–1118 10.1152/physrev.00023.2007 18626067

[B32] Hogue I. B. Hoppe A. Ono A. (2009). Quantitative fluorescence resonance energy transfer microscopy analysis of the human immunodeficiency virus type 1 Gag–Gag interaction: relative contributions of the CA and NC domains and membrane binding. *J. Virol.* 83 7322–7336 10.1128/JVI.02545-08 19403686 PMC2704781

[B33] Inlora J. Chukkapalli V. Derse D. Ono A. (2011). Gag localization and virus-like particle release mediated by the matrix domain of human T-lymphotropic virus type 1 Gag are less dependent on phosphatidylinositol-(45)-bisphosphate than those mediated by the matrix domain of HIV-1 Gag. *J. Virol.* 85 3802–3810 10.1128/JVI.02383-10 21289126 PMC3126146

[B34] Jouvenet N. Neil S. J. Bess C. Johnson M. C. Virgen C. A. Simon S. M. (2006). Plasma membrane is the site of productive HIV-1 particle assembly. *PLoS Biol.* 4:e435 10.1371/journal.pbio.0040435 PMC1750931 17147474

[B35] Jouvenet N. Simon S. M. Bieniasz P. D. (2009). Imaging the interaction of HIV-1 genomes and Gag during assembly of individual viral particles. *Proc. Natl. Acad. Sci. U.S.A.* 106 19114–19119 10.1073/pnas.0907364106 19861549 PMC2776408

[B36] Khorchid A. Halwani R. Wainberg M. A. Kleiman L. (2002). Role of RNA in facilitating Gag/Gag–Pol interaction. *J. Virol.* 76 4131–4137 10.1128/JVI.76.8.4131-4137.2002 11907255 PMC136082

[B37] Konig R. Zhou Y. Elleder D. Diamond T. L. Bonamy G. M. Irelan J. T. (2008). Global analysis of host–pathogen interactions that regulate early-stage HIV-1 replication. *Cell* 135 49–60 10.1016/j.cell.2008.07.032 18854154 PMC2628946

[B38] Krausslich H. G. Facke M. Heuser A. M. Konvalinka J. Zentgraf H. (1995). The spacer peptide between human immunodeficiency virus capsid and nucleocapsid proteins is essential for ordered assembly and viral infectivity. *J. Virol.* 69 3407–3419 7745687 10.1128/jvi.69.6.3407-3419.1995 PMC189053

[B39] Kutluay S. B. Bieniasz P. D. (2010). Analysis of the initiating events in HIV-1 particle assembly and genome packaging. *PLoS Pathog.* 6:e1001200 10.1371/journal.ppat.1001200 PMC2987827 21124996

[B40] Li H. Dou J. Ding L. Spearman P. (2007). Myristoylation is required for human immunodeficiency virus type 1 Gag–Gag multimerization in mammalian cells. *J. Virol.* 81 12899–12910 10.1128/JVI.01280-07 17881447 PMC2169113

[B41] Lindwasser O. W. Resh M. D. (2001). Multimerization of human immunodeficiency virus type 1 Gag promotes its localization to barges, raft-like membrane microdomains. *J. Virol.* 75 7913–7924 10.1128/JVI.75.17.7913-7924.2001 11483736 PMC115035

[B42] Lingwood D. Kaiser H. J. Levental I. Simons K. (2009). Lipid rafts as functional heterogeneity in cell membranes. *Biochem. Soc. Trans.* 37 955–960 10.1042/BST0370955 19754431

[B43] Muriaux D. Costes S. Nagashima K. Mirro J. Cho E. Lockett S. (2004). Role of murine leukemia virus nucleocapsid protein in virus assembly. *J. Virol.* 78 12378–12385 10.1128/JVI.78.22.12378-12385.2004 15507624 PMC525092

[B44] Murray P. S. Li Z. Wang J. Tang C. L. Honig B. Murray D. (2005). Retroviral matrix domains share electrostatic homology: models for membrane binding function throughout the viral life cycle. *Structure* 13 1521–1531 10.1016/j.str.2005.07.010 16216583

[B45] Nadaraia-Hoke S. Bann D. V. Lochmann T. L. Gudleski-O’regan N. Parent L. J. (2013). Alterations in the MA and NC domains modulate phosphoinositide-dependent plasma membrane localization of the Rous sarcoma virus Gag protein. *J. Virol.* 87 3609–3615 10.1128/JVI.03059-12 23325682 PMC3592118

[B46] Naghavi M. H. Goff S. P. (2007). Retroviral proteins that interact with the host cell cytoskeleton. *Curr. Opin. Immunol.* 19 402–407 10.1016/j.coi.2007.07.003 17707624 PMC2040053

[B47] Nguyen D. G. Hildreth J. E. (2003). Involvement of macrophage mannose receptor in the binding and transmission of HIV by macrophages. *Eur. J. Immunol.* 33 483–493 10.1002/immu.200310024 12645947

[B48] Nishi M. Ryo A. Tsurutani N. Ohba K. Sawasaki T. Morishita R. (2009). Requirement for microtubule integrity in the SOCS1-mediated intracellular dynamics of HIV-1 Gag. *FEBS Lett.* 583 1243–1250 10.1016/j.febslet.2009.03.041 19327355

[B49] Nydegger S. Foti M. Derdowski A. Spearman P. Thali M. (2003). HIV-1 egress is gated through late endosomal membranes. *Traffic* 4 902–910 10.1046/j.1600-0854.2003.00145.x 14617353

[B50] O’Carroll I. P. Crist R. M. Mirro J. Harvin D. Soheilian F. Kamata A. (2012). Functional redundancy in HIV-1 viral particle assembly. *J. Virol.* 86 12991–12996 10.1128/JVI.06287-11 22993163 PMC3497692

[B51] O’Carroll I. P. Soheilian F. Kamata A. Nagashima K. Rein A. (2013). Elements in HIV-1 Gag contributing to virus particle assembly. *Virus Res.* 171 341–345 10.1016/j.virusres.2012.10.016 23099087 PMC6771925

[B52] Ono A. (2009). HIV-1 assembly at the plasma membrane: Gag trafficking and localization. *Future Virol.* 4 241–257 10.2217/fvl.09.4 19802344 PMC2676728

[B53] Ono A. Ablan S. D. Lockett S. J. Nagashima K. Freed E. O. (2004). Phosphatidylinositol (45) bisphosphate regulates HIV-1 Gag targeting to the plasma membrane. *Proc. Natl. Acad. Sci. U.S.A.* 101 14889–14894 10.1073/pnas.0405596101 15465916 PMC522033

[B54] Ono A. Orenstein J. M. Freed E. O. (2000). Role of the Gag matrix domain in targeting human immunodeficiency virus type 1 assembly. *J. Virol.* 74 2855–2866 10.1128/JVI.74.6.2855-2866.2000 10684302 PMC111776

[B55] Ootsuyama Y. Shimotohno K. Miwa M. Oroszlan S. Sugimura T. (1985). Myristylation of gag protein in human T-cell leukemia virus type-I and type-II. *Jpn. J. Cancer Res.* 76 1132–1135 3005204

[B56] Oroszlan S. Copeland T. D. (1985). Primary structure and processing of gag and env gene products of human T-cell leukemia viruses HTLV-ICR and HTLV-IATK. *Curr. Top. Microbiol. Immunol.* 115 221–233 10.1007/978-3-642-70113-9_14 2983943

[B57] Pelchen-Matthews A. Kramer B. Marsh M. (2003). Infectious HIV-1 assembles in late endosomes in primary macrophages. *J. Cell Biol.* 162 443–455 10.1083/jcb.200304008 12885763 PMC2172706

[B58] Perez-Caballero D. Hatziioannou T. Martin-Serrano J. Bieniasz P. D. (2004). Human immunodeficiency virus type 1 matrix inhibits and confers cooperativity on gag precursor–membrane interactions. *J. Virol.* 78 9560–9563 10.1128/JVI.78.17.9560-9563.2004 15308748 PMC506924

[B59] Purohit P. Dupont S. Stevenson M. Green M. R. (2001). Sequence-specific interaction between HIV-1 matrix protein and viral genomic RNA revealed by in vitro genetic selection. *RNA* 7 576–584 10.1017/S1355838201002023 11345436 PMC1370111

[B60] Qualley D. F. Stewart-Maynard K. M. Wang F. Mitra M. Gorelick R. J. Rouzina I. (2010). C-terminal domain modulates the nucleic acid chaperone activity of human T-cell leukemia virus type 1 nucleocapsid protein via an electrostatic mechanism. *J. Biol. Chem.* 285 295–307 10.1074/jbc.M109.051334 19887455 PMC2804176

[B61] Raposo G. Moore M. Innes D. Leijendekker R. Leigh-Brown A. Benaroch P. (2002). Human macrophages accumulate HIV-1 particles in MHC II compartments. *Traffic* 3 718–729 10.1034/j.1600-0854.2002.31004.x 12230470

[B62] Rayne F. Bouamr F. Lalanne J. Mamoun R. Z. (2001). The NH2-terminal domain of the human T-cell leukemia virus type 1 capsid protein is involved in particle formation. *J. Virol.* 75 5277–5287 10.1128/JVI.75.11.5277-5287.2001 11333909 PMC114933

[B63] Rein A. Datta S. A. Jones C. P. Musier-Forsyth K. (2011). Diverse interactions of retroviral Gag proteins with RNAs. *Trends Biochem. Sci.* 36 373–380 10.1016/j.tibs.2011.04.001 21550256 PMC3130074

[B64] Resh M. D. (1999). Fatty acylation of proteins: new insights into membrane targeting of myristoylated and palmitoylated proteins. *Biochim. Biophys. Acta* 1451 1–16 10.1016/S0167-4889(99)00075-0 10446384

[B65] Resh M. D. (2004). A myristoyl switch regulates membrane binding of HIV-1 Gag. *Proc. Natl. Acad. Sci. U.S.A.* 101 417–418 10.1073/pnas.0308043101 14707265 PMC327161

[B66] Sonnino S. Prinetti A. (2013). Membrane domains and the “lipid raft” concept. *Curr. Med. Chem.* 20 4–21 10.2174/0929867311320010003 23150999

[B67] Stansell E. Apkarian R. Haubova S. Diehl W. E. Tytler E. M. Hunter E. (2007). Basic residues in the Mason-Pfizer monkey virus gag matrix domain regulate intracellular trafficking and capsid–membrane interactions. *J. Virol.* 81 8977–8988 10.1128/JVI.00657-07 17596311 PMC1951391

[B68] Tang C. Loeliger E. Luncsford P. Kinde I. Beckett D. Summers M. F. (2004). Entropic switch regulates myristate exposure in the HIV-1 matrix protein. *Proc. Natl. Acad. Sci. U.S.A.* 101 517–522 10.1073/pnas.0305665101 14699046 PMC327179

[B69] Vale R. D. (2003). The molecular motor toolbox for intracellular transport. *Cell* 112 467–480 10.1016/S0092-8674(03)00111-9 12600311

[B70] van Meer G. Voelker D. R. Feigenson G. W. (2008). Membrane lipids: where they are and how they behave. *Nat. Rev. Mol. Cell Biol.* 9 112–124 10.1038/nrm2330 18216768 PMC2642958

[B71] Vogt V. M. Simon M. N. (1999). Mass determination of rous sarcoma virus virions by scanning transmission electron microscopy. *J. Virol.* 73 7050–7055 10400808 10.1128/jvi.73.8.7050-7055.1999 PMC112795

[B72] von Schwedler U. K. Stray K. M. Garrus J. E. Sundquist W. I. (2003). Functional surfaces of the human immunodeficiency virus type 1 capsid protein. *J. Virol.* 77 5439–5450 10.1128/JVI.77.9.5439-5450.2003 12692245 PMC153941

[B73] Welsch S. Muller B. Krausslich H. G. (2007). More than one door–budding of enveloped viruses through cellular membranes. *FEBS Lett.* 581 2089–2097 10.1016/j.febslet.2007.03.060 17434167 PMC7126970

[B74] Yeager M. Wilson-Kubalek E. M. Weiner S. G. Brown P. O. Rein A. (1998). Supramolecular organization of immature and mature murine leukemia virus revealed by electron cryo-microscopy: implications for retroviral assembly mechanisms. *Proc. Natl. Acad. Sci. U.S.A.* 95 7299–7304 10.1073/pnas.95.13.7299 9636143 PMC22596

[B75] Zhou H. Xu M. Huang Q. Gates A. T. Zhang X. D. Castle J. C. (2008). Genome-scale RNAi screen for host factors required for HIV replication. *Cell Host Microbe* 4 495–504 10.1016/j.chom.2008.10.004 18976975

[B76] Zhou W. Parent L. J. Wills J. W. Resh M. D. (1994). Identification of a membrane-binding domain within the amino-terminal region of human immunodeficiency virus type 1 Gag protein which interacts with acidic phospholipids. *J. Virol.* 68 2556–2569 8139035 10.1128/jvi.68.4.2556-2569.1994 PMC236733

[B77] Zhou W. Resh M. D. (1996). Differential membrane binding of the human immunodeficiency virus type 1 matrix protein. *J. Virol.* 70 8540–8548 8970978 10.1128/jvi.70.12.8540-8548.1996 PMC190946

